# Comparison of West African and Congo Basin Monkeypox Viruses in BALB/c and C57BL/6 Mice

**DOI:** 10.1371/journal.pone.0008912

**Published:** 2010-01-27

**Authors:** Christina L. Hutson, Jason A. Abel, Darin S. Carroll, Victoria A. Olson, Zachary H. Braden, Christine M. Hughes, Michael Dillon, Consuelo Hopkins, Kevin L. Karem, Inger K. Damon, Jorge E. Osorio

**Affiliations:** 1 Centers for Disease Control and Prevention, Atlanta, Georgia, United States of America; 2 Department of Pathobiological Sciences, School of Veterinary Medicine, University of Wisconsin, Madison, Wisconsin, United States of America; University of Pretoria, South Africa

## Abstract

Although monkeypox virus (MPXV) studies in wild rodents and non-human primates have generated important knowledge regarding MPXV pathogenesis and inferences about disease transmission, it might be easier to dissect the importance of virulence factors and correlates of protection to MPXV in an inbred mouse model. Herein, we compared the two clades of MPXV via two routes of infection in the BALB/c and C57BL/6 inbred mice strains. Our studies show that similar to previous animal studies, the Congo Basin strain of MPXV was more virulent than West African MPXV in both mouse strains as evidenced by clinical signs. Although animals did not develop lesions as seen in human MPX infections, localized signs were apparent with the foot pad route of inoculation, primarily in the form of edema at the site of inoculation; while the Congo Basin intranasal route of infection led to generalized symptoms, primarily weight loss. We have determined that future studies with MPXV and laboratory mice would be very beneficial in understanding the pathogenesis of MPXV, in particular if used in *in vivo* imaging studies. Although this mouse model may not suffice as a model of human MPX disease, with an appropriate inbred mouse model, we can unravel many unknown aspects of MPX pathogenesis, including virulence factors, disease progression in rodent hosts, and viral shedding from infected animals. In addition, such a model can be utilized to test antivirals and the next generation of orthopoxvirus vaccines for their ability to alter the course of disease.

## Introduction

Human monkeypox (MPX) is a sporadic smallpox-like zoonotic viral exanthemata disease that occurs in the rain forests of Central and West Africa. The virus was discovered in 1958 from primate tissues[1]. The disease has similar manifestations to those of smallpox but tends to be less severe. The virus MPX belongs to the *Orthopoxvirus* genus of *Poxviridae*, and shares many biochemical and physical properties with other orthopoxviruses, such as vaccinia and variola (causative agent of smallpox). MPXV is endemic in the rain forests of Central and West Africa, causing sporadic outbreaks in remote villages, where it is believed to result from close contact between humans and animals living in the rain forests[2]–[4]. MPXV infection is thought to be acquired most commonly by direct percutaneous contact, mucosal or respiratory exposure to tissues or fluids of infected animals[5].

An outbreak of MPXV recently occurred in the US in 2003[6]–[9]. Imported African rodents were implicated as the source of the outbreak. Studies have demonstrated the existence of two genetically distinct variants of the virus: West African and Congo Basin clades[10], [11]. The strain that caused the US outbreak belonged to the West African clade, which clinical and epidemiological data has shown is associated with less severe disease[10].

Several animal models have been used to study MPXV pathogenesis including rabbits and newborn white mice[12], cynomolgus monkeys[13], squirrels[14]–[16], prairie dogs[16], [17] and dormice[18]. While studies in wild rodents and non-human primates have generated important knowledge regarding MPXV pathogenesis and inferences about disease transmission, it might be easier to dissect the importance of virulence factors and correlates of protection to MPXV in an inbred mouse model. Laboratory mouse models are very versatile, allowing the roles of individual components of innate and adaptive immunity to be investigated through well-characterized inbred strains as well as gene deficient mice. With an inbred mouse model, we can investigate many unknown aspects of MPXV pathogenesis, including: virulence factors, disease progression in rodent hosts, and viral shedding from infected animals; an index of the transmission potential to humans and other animals. In addition, such a model can be utilized to test anti-virals and the next generation of orthopoxvirus vaccines for their ability to alter the course of disease.

## Materials and Methods

### Ethics Statement

All animals were handled in strict accordance with good animal practice as defined by the relevant national and/or local animal welfare bodies, and all animal work was approved by the CDC Institutional Animal Care and Use Committee (IACUC) under an approved protocol (1457REGMOUC).

### Animal Maintenance

Inbred C57BL/6 and BALB/c mice (6–7 weeks-old) were obtained from Harlan Sprague Dawley (Indianapolis, IN). During experimental infections animals were housed individually in mouse cages with aerosol filter tops and kept in a Duo-Flow biosafety cabinet (Lab Products, Inc.) in the Biosafety Level 3 (BSL-3) animal facility at the CDC. All animal studies were conducted in the Biosafety Level 3 (BSL-3) animal facility at the CDC and approved by the CDC Institutional Animal Care and Use Committee (IACUC) under an approved protocol (1457REGMOUC).

### Viruses

The West African strain of MPXV used in these studies had been isolated during the 2003 U.S. outbreak and is designated as MPXV-2003-044 and has previously been fully sequenced and identified as a West African MPXV strain[11]. This isolate is from a prairie dog associated with the index case in Wisconsin during the U.S. outbreak[11], [19]. The MPXV collected from the Congo Basin was isolated during a 2003 outbreak in the Republic of Congo (ROC) and is designated as MPXV-2003-358. The later isolate was from a 10-year-old girl infected in Impfondo, ROC, and has also been fully sequenced and thus characterized as a Congo Basin clade representative MPXV[11]. Both viruses had undergone two passages in African green monkey kidney cells (BSC-40) prior to seed pool production; purified preparations of virus were used for animal challenges.

### Inoculation of Animals

Groups of five BALB/c and C57BL/6 mice were inoculated via subcutaneous injection in the footpad (FP) or via an intranasal (IN) route with either the West African or Congo Basin MPXV strain (10^5^ p.f.u. in a total volume of 10 µl per mouse, similar to an inoculum the author's have used in a previous study). In addition, two mice of each mouse strain were mock infected with PBS via the FP or IN route. Decontamination of all equipment, surfaces, gloves, etc. with 20% Lysol ® followed by 10% ethanol was done between strains to alleviate the chance of cross-contamination. Isoflurane-soaked cotton balls placed in 50 ml conical tubes were used to anesthetize mice. The appropriate virus was then administered with an eppendorf pipette into the nostrils (5 µl into each nostril) for the IN route. For the FP route, the appropriate virus (10 µl) was administered into the right footpad by subcutaneous injection using a 28-gauge needle. Care was taken to avoid the intramuscular and/or intradermal tissue.

### Sampling, Necropsy and Tissue Specimen Collection

Overall activity and clinical observations were made on a daily basis. Animals were weighed individually every week, or more often if needed. Based on what the author's have seen in previous studies with prairie dogs[17], animals were sampled for 30 days after infection to allow full recoveries from viral infection. At day 30 post infection (p.i.), animals were humanely euthanized and blood, urine, feces and swabs from oral, nasal, and rectal orifices were collected from all animals. Swabs were collected with sterile individual Dacron swabs and stored frozen without diluents. Serum was separated from whole blood collected by either nobuto strips (Advantec MFS, Inc.) or direct cardiac sticks after euthanasia. Mice were humanely euthanized and quickly frozen at −70°C. The bodies were subsequently thawed and necropsies were performed utilizing full BSL-3 personal protective equipment. Samples collected at necropsy included brain, heart, lung, spleen, liver, kidney, tongue, lymph nodes, gonad, and skin. Between sample collections, all instruments (scalpel, scissors, forceps, etc.) were cleaned and decontaminated using 3% Amphyll and 10% Clorox bleach solutions and then rinsed in sterile water. Tissues and swabs were frozen at −70°C prior to further processing for both DNA analysis and virus isolation (see below).

### Sample Preparation

All sample processing was performed under BSL-3 conditions. For swabs, 400 µl of PBS was added. The Swab extraction tube systems (SETS) (Roche) protocol was used to recover swab samples. 100 µl from swabs or blood samples were used to extract genomic DNA using the AquaPure DNA isolation kit (Bio-Rad). The remaining swab lysate was used for virus isolation (see below). Tissue and fecal samples placed in disposable dounce homogenizers with 1.0 ml of PBS and the tissue was homogenized thoroughly. About 100 ml of the homogenized sample was used to extract genomic DNA (AquaPure DNA isolation kit) and the remaining volume was used for virus isolation (see below).

### Real-Time PCR Analysis

Samples were tested by real-time PCR using forward and reverse primers and probe which are complimentary to regions of the well conserved E9L (DNA polymerase) gene of orthopoxviruses[20]. Serial 10-fold dilutions of MPXV DNA (10 fg–1 ng) were used as a positive controls. Reactions were placed in an ABI 7900 or 7900HT real time PCR detector (Applied Biosystems) and subjected to the following thermal cycle parameters: 95°C for 10 minutes, then 95°C for 15 seconds and 63°C for 1 minute for 45 cycles. A sample was considered positive if the cycle threshold (Ct) value for both duplicates was 37 or below. A weak positive was a sample with Ct values of 38–39. PCR samples were called inconclusive if only one of the duplicate samples tested positive or weakly positive.

### Virus-Tissue Infectivity

To screen tissues for the presence of MPX virus, previous analysis has demonstrated that real-time PCR detection of MPXV DNA was significantly more sensitive than the detection of viable virus (plaque forming units)[6]. For the current study, specimens were first tested for the presence of orthopoxvirus (OPXV) DNA by PCR and, if positive, they were subsequently evaluated for viable virus by tissue culture inoculation. Each swab, feces or individual tissue sample to be assayed was titrated using 10-fold dilutions of swab sample or tissue homogenate in a standard OPXV plaque assay on BSC-40 cell monolayers, incubated at 36°C, 6% CO_2_ for 72 hours, and subsequently stained with 2X crystal violet stain and formalin to reveal plaques.

### Serologic Analysis

As previously described[17], a modified ELISA was used for analysis of anti-*Orthopoxvirus* immunoglobulin types A and G. Because of the small amount of blood collected from each animal, mouse sera were pooled for each group. Pooled sera samples were added to both halves of the plates at a dilution of 1∶100 in assay diluent. Optical densities (ODs) were read on a spectrophotometer at 450 nm. Values reported represented the average of duplicate wells for each sample. Both positive and negative human anti-vaccinia sera were used as assay controls. The BSC-40 cell lysate half of each plate was used to generate a cut-off value (COV) for each plate by averaging all the values of the BSC-40 lysate half and adding two standard deviations. Specimens were considered positive if the test sample's value was above the COV.

### Statistical Analyses

Nonparametric statistical tests were used in the analysis as data were not normally distributed[21]. Comparisons of weight loss between strains, routes of infection, and mouse type were made using the Wilcoxon rank-sum test. In order to evaluate weight differences, day zero weights were used as the baseline and the lowest weight measured thereafter was used to calculate percent weight loss in each animal. A p-value of <0.05 was considered significant.

## Results

### Congo Basin FP

**Figure 1:**
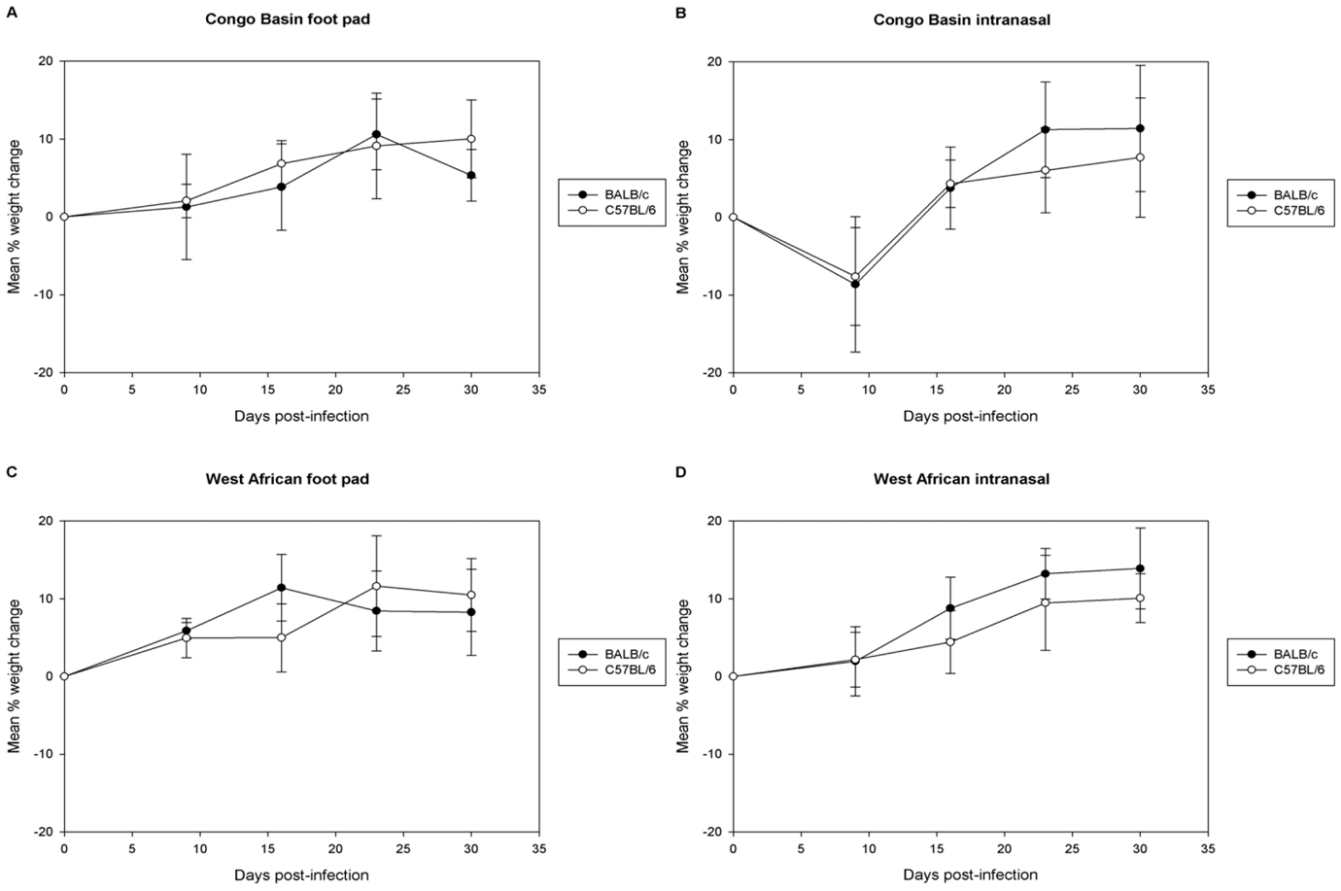
Mean percent weight change comparison of mice infected with MPXV. BALB/c and C57BL/6 mice were challenged with either Congo Basin MPXV (A, B) or West African MPXV (C, D) by either an intranasal (n = 5) or foot pad (n = 5) route of infection.

Until day 6 p.i., no overt signs of disease were noted for any animal. Beginning on day 6 all mice inoculated in the FP with the Congo Basin MPXV had noticeable edema at the site of inoculation, compared to control FP mice, which showed no signs of edema at any time throughout the study. BALB/c mice had greater edema compared to C57BL/6 mice (Table 1). By day 7 p.i., one of the five C57BL/6 mice and two of the five BALB/c mice had a FP severely swollen and affected animals were not putting any pressure on the appendage. One of these BALB/c mice also had a ruffled hair coat, and the right leg and lymph node appeared to be swollen. On day 9, all C57BL/6 mice still had a slightly swollen FP, but none of these animals were favoring the foot. In contrast, BALB/c FP animals were still significantly swollen, with one mouse limping. By day 13 p.i., edema had resolved in all the FPs and all mice were walking normally. None of the C57BL/6 FP inoculated mice lost weight throughout the course of the study (figure 1). Of the BALB/c mice, 1 of 5 lost 7.3% of its starting body weight between day 0 and day 9. All of the mice inoculated in the FP with Congo Basin MPXV survived infection and were euthanized on day 30 p.i., at which time representative samples were taken for testing. Two BALB/c mice and one C57BL/6 mouse had positive or inconclusive PCR samples (Table 1). All PCR positive and inconclusive samples were titrated, however none yielded viable virus (Table 1).

**Table 1:** Comparison of descriptive disease presentation and molecular findings in mice infected with monkeypox virus (MPXV).

	West African MPXV		Congo Basin MPXV	
	C57BL/6	BALB/c	C57BL/6	BALB/c
**Foot pad edema**	5/5 FP¶0/5 IN	5/5 FP¶0/5 IN	5/5 FP†0/5 IN	5/5 FP†0/5 IN
**Limping**	5/5 FP0/5 IN	2/5 FP0/5 IN	5/5 FP*0/5 IN	5/5 FP*0/5 IN
**Weight loss**	0/5 FP0/5 IN	0/5 FP0/5 IN	0/5 FP4/5 IN	1/5 FP3/5 IN
**Ruffled hair coat**	0/5 FP0/5 IN	1/5 FP0/5 IN	0/5 FP0/5 IN	1/5 FP4/5 IN
**Positive PCR animals**	1/5 FP1/5 IN‡	1/5 inc FP3/5 IN	1/5 inc FP2/5 inc IN	2/5 FP‡0/5 IN
**Positive and inconclusive PCR samples**	FP: LiIN: LN, Sk,RS-inc	FP: Lu-incIN: (1) Lu(2) BL, Ki-inc(3) LN	FP: Sp-incIN: Sp-X 2 inc	FP: (1) Li, Lu, Ki(2) Lu-inc
**OPXV antibodies**	FP: posIN: pos	FP: posIN: pos	FP: posIN: pos	FP: posIN: weak pos

C57BL/6 and BALB/c mice were challenged with either Congo Basin MPXV or West African MPXV by an intranasal (IN) or foot pad (FP) route of infection (n = 5 in each group). Samples for PCR and serology were taken at 30 days post infection.

¶ Mild swelling noted.

† Severe swelling noted.

* Severe limping with some animals putting little/no weight on limb.

‡ More than 1 sample positive or inconclusive.

Inc: Inconclusive PCR sample.

Positive/Inconclusive PCR samples: Li (liver), LN (lymph node), Sk (skin), RS (rectal swab), Lu (lung), BL (blood), Ki (kidney), Sp (spleen).

### Congo Basin IN

As was seen with the Congo Basin FP model, no observable symptoms were noted for the first 6 days p.i. Four of five BALB/c mice inoculated IN with the Congo Basin MPXV showed a ruffled coat on days 6–9 compared to none of IN infected C57BL/6 mice. However, weight loss was observed in both strains of mice (Figure 1). Four of the five C57BL/6 mice had weight loss between days 0–9 ranging between 7.3–14% starting body weight (Mean = 8.16%). Similarly, 3 of the 5 BALB/c mice also had weight loss between days 0–9, ranging between 3–19% starting body weight (Mean = 9.14%). Otherwise, no obvert signs of morbidity (i.e., lesions) were observed in the IN Congo Basin inoculated mice. As was seen with the Congo FP group, all of the mice survived infection and were euthanized on day 30 p.i., at which time representative samples were taken for testing. Two C57BL/6 mice had inconclusive PCR spleen samples, both of which were negative for viable virus. None of the BALB/c mice had MPXV PCR positive or inconclusive samples.

### West African FP

Similar to the Congo Basin animals, until day 6 p.i., no overt signs of disease were noted for any mice in this group. Only one out of five BALB/c mice was slightly favoring the inoculated foot and had a ruffled coat on day 6. However, beginning on day 7 p.i., all mice inoculated in the FP with West African MPXV had slight edema at the site of inoculation. The edema that was observed in the West African FP inoculated mice was comparatively less than that observed in the Congo Basin FP animals. Additionally, by day 9 for the C57BL/6 mice, and day 11 for the BALB/c mice, the edema had resolved completely. This was slightly earlier than that seen with the Congo Basin MPXV FP animals. None of the West African FP inoculated mice lost weight throughout the course of the study or developed any lesions. As was seen for both Congo Basin inoculation routes, all animals in this group survived infection and were euthanized 30 days p.i. with representative samples collected for testing. One BALB/c mouse had the lung sample test inconclusive and one C57BL/6 mouse had a positive lung sample for MPXV DNA. However, neither of these samples was positive for viable virus.

### Western African IN

Unlike the Congo Basin IN challenged animals, none of the mice in the West African MPXV IN inoculated group developed any obvious signs of morbidity. All animals survived infection and were euthanized 30 days p.i., with representative samples collected for testing. Although no clinical symptoms were observed, some samples yielded PCR positive results (Table 1), however none yielded viable virus.

### Control Mice

**Figure 2:**
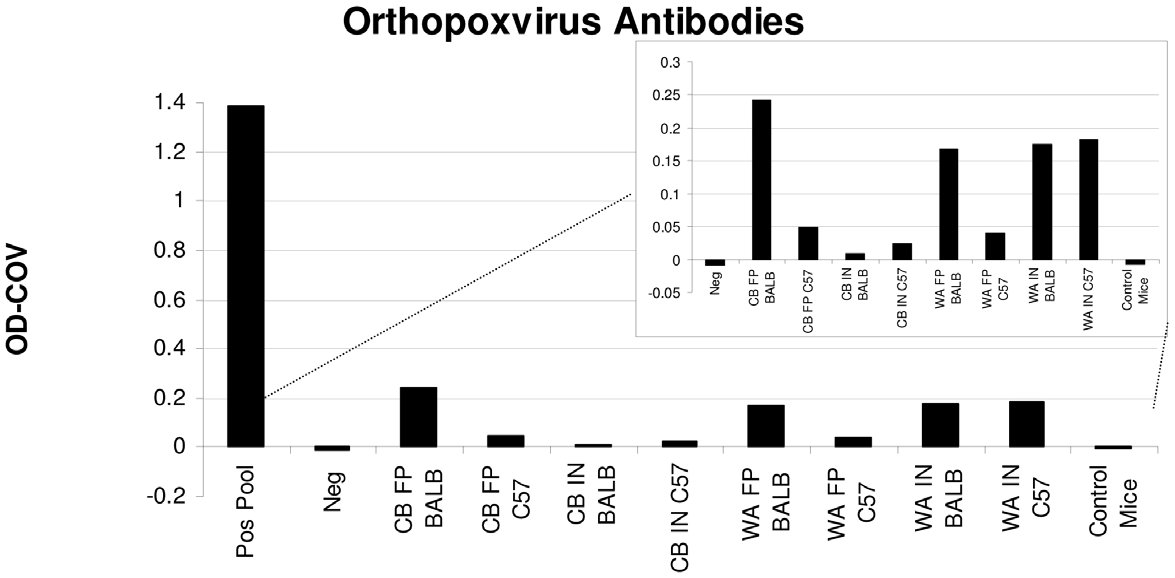
Serologic analysis of mice infected with MPXV. BALB/c and C57BL/6 mice were challenged with either Congo Basin MPXV (CB) or West African MPXV (WA) by either an intranasal (IN) or foot pad (FP) route of infection (n = 5 in each group). Additionally, 4 mice were mock infected with PBS (controls). Because of the small amount of blood collected, mouse sera were pooled from each group of animals for testing. Data are shown with the positive controls as well as without positive controls (inset) in order to better visualize samples with low levels of antibodies.

None of the four control, mock-infected mice showed any signs of illness (i.e., edema, weight loss, lesions). Furthermore, all necropsy samples from these control mice tested negative for MPXV DNA and all four animals were negative for OPXV antibodies (Figure 2).

### Serology

Pooled samples from the groups of mice collected at time of necropsy were used for serology. At a 1∶100 dilution, all groups were considered positive for OPXV antibodies on the day of necropsy, day 30 p.i. (Figure 2). Because of the small amounts of serum used and the different collection methods, we can not compare the serology results between groups, except to confirm that all seroconverted.

## Discussion

In the present study, we compared the infection produced by West African and Congo Basin MPXV clades in inbred BALB/c and C57BL/6 mice. Subtle differences in pathogenicity between the two strains of MPXV were observed in this study. As seen in previous animal studies[10], [17], the Congo Basin strain of MPXV was more virulent than the West African strain as evidenced by the symptoms we observed. We have determined that laboratory mice may be a suitable animal model to study MPXV pathogenesis and future studies to further characterize the mouse innate and adaptive immune responses to monkeypox infection will help in understanding the host-virus interactions that lead to more attenuated disease presentations compared to people and other animals such as prairie dogs.

Localized signs and symptoms were more noticeable in the FP model, whereas generalized signs and symptoms were more evident in the IN challenge models. On the whole, for both mouse strains and both inoculation routes, the West African MPXV disease was less severe than that observed in the Congo Basin challenged groups. Although FP edema was observed in all mice that were FP inoculated, the edema observed in the West African infected group was comparatively less (no/slight limp) than that observed in the Congo Basin FP mice (severe limp with animal putting little/no weight on inoculated leg). Furthermore, the edema resolved in a shorter time period in the West African MPXV infected mice compared to the Congo Basin MPXV infected mice. Additionally, one of the Congo Basin FP animals lost weight, whereas none of the West African FP animals lost weight. When we compare the IN route of infection, unlike the Congo Basin IN animals which displayed some ruffled coats and weight loss, the animals in the West African IN group did not develop any noticeable symptoms. Both mouse strains that were challenged IN with Congo Basin MPXV had an observed trend of increased weight loss compared to West African MPXV challenged mice. However the weight loss was not considered statistically significant (C57BL/6 mice: mean % weight loss = 0.56% West African vs. 8.16% Congo Basin, p = 0.076; BALB/c mice: mean % weight loss = 1.08% West African vs. 9.14% Congo Basin, p = 0.099). However, utilizing the blood collection methods in this study, the immune response observed in the Congo Basin IN challenged mice was less than that observed in the West African IN challenged mice. Future studies on tracking virus during the course of infection may help to clarify this apparent difference in viral clearance by or virus presentation to the immune system.

The edema that occurred in the FP groups with both strains of MPXV suggests an acute inflammation. The observation that only mild ruffled coats and weight loss was seen in the Congo Basin IN animals and no observable symptoms in the West African IN animals could imply more of a generalized inflammatory response to infection in the Congo Basin IN infected animals. Alternatively, the weight loss that was seen in the Congo Basin IN animals may have been caused by localized inflammation in the nasal and oral cavities of these Congo Basin IN challenged animals resulting in decreased food consumption. However, the findings that all MPXV challenged groups had seroconverted at time of necropsy suggests that the animals did develop infection. Not surprisingly and as previously seen in the authors' studies with prairie dogs[17], no viable virus was found in necropsy samples at 30 days p.i. However, additional evidence of systemic infection is provided by the positive PCR results in several tissue types including lungs, blood, lymph node, skin, liver and kidney samples at 30 days p.i. Although PCR positive tissue samplings were not found at day 30 in the Congo Basin IN challenged animals, in the West African MPXV IN challenged animals in which no symptoms were observed, at 30 days p.i. animals had several positive PCR samples suggestive of systemic infection.

Although all mice in the present study were considered OPXV antibody positive, it is noteworthy that the antibody levels seen in these animals were less than what we have observed when challenging prairie dogs with a comparable inoculums of MPXV[17]. Although these ELISA plates were run on different days, because our negative and positive controls are similar throughout ELISA runs it is possible to compare the data. The low levels of antibodies detected in the mouse sera could reflect the degree of infectious virus that is presented to the immune system in these inbred mouse strains. Future studies may better address the mouse innate and adaptive immune responses to monkeypox infection, and will help in understanding the host-virus interactions that lead to more attenuated disease presentations.

Inbred mice are as genetically alike as possible, being homozygous at virtually all of their loci. Such genetic homogenous models are ideal for studying human pathogens as symptoms of disease are very similar or identical between animals. Results therefore are often more easily interpreted than in a wild or out bred animal model in which genetic diversity can sometimes lead to conflicting or confusing results. Inbred mice, including BALB/c and C57BL/6 strains, are a very controlled and well understood animal model and specific commercial reagents have been created for these animals. It would also be possible to create knockout mice to further understand and characterize the relationship between the immune system and the virus. Having such an inbred mouse model for the study of MPXV could greatly increase our wealth of knowledge about this serious human pathogen.

A recent paper by co-authors of this manuscript [22] used *in vivo* imaging to study IP (intraperitoneal) inoculation of monkeypox in immunocompetent and immuno-compromised BALB/c mice. Although the IP route of infection does not mimic the natural transmission of this disease, the previous results were similar to our study in that the innate immune system in the immunocompetent animals kept the virus localized to the site of infection while the immuno-compromised animals developed systemic infections. Also, in both groups of animals in the previous study, the Congo Basin strain of monkeypox was more virulent which is also congruent with our results. Future *in vivo* imaging studies with an IN route of infection would better mimic natural infection routes, and would provide a better understanding of “natural” patterns viral trafficking and the immune response(s) that protect against MPX infection in immunocompetent animals.

We have demonstrated that these mouse strains would not likely suffice as a model for human MPX disease due to the more subtle disease presentation compared to the disease course seen in humans, but may prove informative in the understanding of immune response which makes certain animals more capable of efficiently clearing MPXV. Both mice strains had similar clinical presentations, with the exception of more BALB/c mice developing ruffled coats when challenged with Congo Basin MPXV. Therefore, the BALB/c mouse strain should probably be used in future studies. However, based on symptoms of morbidity, the Congo Basin MPXV clade was more virulent in both strains of mice. The FP inoculation route provided the visual symptom of edema with both MPXV strains. However, the animals challenged IN with Congo Basin MPXV lost weight and 4/5 BALB/c mice developed ruffled coats unlike West African IN challenged animals, suggesting that the IN route might be better for clade comparison. Additionally, as previously mentioned, the IN route is the more probable natural route of infection, and therefore will be used in future studies. In particular, this mouse model may be used for non-invasive *in vivo* imaging in order to unravel many unknown aspects of MPX pathogenesis, including virulence factors, disease progression in rodent hosts, and viral shedding from infected animals. Furthermore, an *in vivo* inbred mouse model could be used to test antivirals and the next generation of OPXV vaccines for their ability to alter the course of monkeypox disease.
